# An empirical study on spatial–temporal dynamics and influencing factors of apple production in China

**DOI:** 10.1371/journal.pone.0240140

**Published:** 2020-10-07

**Authors:** Qiangqiang Zhang, Fanji Shi, Nazir Muhammad Abdullahi, Liqun Shao, Xuexi Huo

**Affiliations:** 1 College of Economics and Management, Northwest A&F University, Yangling, Shannxi, China; 2 Center of Western Rural Development, Northwest A&F University, Yangling, Shannxi, China; 3 Institute of Geographic Sciences and Natural Resources Research, University of Chinese Academy of Sciences, Beijing, China; Murdoch University, AUSTRALIA

## Abstract

In the context of supply-side structural reform, revealing the characteristics of spatial–temporal dynamics and influencing factors of China’s apple production layout is of great significance to ensure apple supply and demand balance and timely adjustment of industrial policies and regional layout strategies. Based on national and provincial apple production data from 1978 to 2016, this study used the apple production concentration index to analyse the evolution characteristics of regional apple production patterns in China. A theoretical analysis framework was established and a spatial econometric model was used to quantitatively explore the influencing factors of China’s apple production layout. The results showed that, first, since the reform and opening-up policy, a general trend of fluctuating growth was found for apple production in China. The centre of apple production layout moved in the southwest direction, with the shift from the Bohai Bay region to the Loess Plateau region. Second, apple production had a significant spatial correlation, while the degree of spatial agglomeration gradually decreased. Third, these changes were significantly influenced by apple comparative income, infrastructure, policies, and climatic conditions. Therefore, it is necessary to continue optimizing and adjusting the apple spatial layout to enhance the technological progress and economic effect of the apple industry and to ensure the stability and balance of regional supply and demand.

## Introduction

Since the reform and opening up policy, the apple has taken the lead in entering market-oriented reform. It was also among the first batch of agricultural products that China planned to fully liberalize in the 1980s [1]. According to the statistics data from the National Bureau of Statistics of China (NBSC) [2, 3], in the era of rising fruit consumption by both urban and rural residents, the area of apple production increased from 0.68 million ha in 1978 to 2.32 million ha in 2016, with an average annual growth rate of 3.29 percent. Meanwhile, the output from apple production has increased from 2.28 million tons in 1978 to 43.88 million tons in 2016, with an 8.10 percent average annual growth rate. In 2016, the areas under apple production and apple output in China accounted for 46.09 percent and 47.41 percent of the global area and output of apple production, respectively. This showed that the area and output in China had increased sharply from 17.30 percent and 7.06 percent in 1978. Thus, China has become the world’s largest apple producer [4, 5]. However, with an increase in income and the improved standard of living among urban and rural residents, consumers are paying more attention to the quality and safety of agricultural products as well as the nutritional value of foods [6–9], thereby accelerating the upgrade of apple market demand structure [10–14]. The spill-over effects of high relative prices of apple, policy support for industrial poverty alleviation, and the heterogeneity of farmers’ individual rationality induced the blind production and expansion of apple production areas in unsuitable and sub-suitable areas. As a consequence, the proportion of apple production areas in unsuitable and sub-suitable areas is too high, the quality and price of apple are gradually declining, and the spatial development of apple production layout is uneven and uncoordinated. The existing apple production and supply model is struggling to adapt to changes in market demand in the new era. In addition, the development of the apple industry faced an imbalance structure between supply and quality structure [15]. Domestic labour costs have risen rapidly, driving up production costs of labour-intensive agricultural products, such as apple. Moreover, the increasing apple import volume of China has produced a crowding-out effect on the domestic apple supply market. The continued growth of domestic apple production has further strengthened the internal and external pressures of the domestic apple industry and forced apple production to improve in quality and efficiency. Driven by high output, high import, and high cost of production, the pressure of structural reform on the supply side of the apple industry is highlighting the necessity and urgency to optimize the spatial layout of China’s apple production.

The issue of the spatial layout of China’s apple production has attracted widespread attention from the Chinese government and scholars. To adjust the production structure of apple and to optimize the regional industrial layout, the Ministry of Agriculture and Rural Affairs (MARA) of the People’s Republic of China has successively formulated a series of policy plans, such as *The Regional Layout Planning of Dominant Agricultural Products (2003–2007)*, *The Advantageous Regional Development Plan for Apple (2003–2007) The Regional Layout Planning of National Advantage Agricultural Products (2008–2015)*, and *Apple Advantage Regional Planning (2008–2015)*. From the academic point of view, studies on apple production layout mainly focus on the following aspects. First, some studies analysed changes in the apple production layout of China, e.g. Liu and Fan [16] and Zhang et al. [17], who found that the apple production layout of China has gradually changed from the four main producing areas of the Bohai Bay, the Loess Plateau, the Yellow River Old Road, and the Southwest Cold and Highland to the two advantageous areas of the Bohai Bay and the Loess Plateau, and the dominant apple-producing areas transferred to the northwest. Second, the literatures addressing the change mechanism of apple production layout in China used the panel data models to reveal that the endogenous competition effect of inter-regional apple production [18], changes in labour and land cost [19–22], the ecological factors of apple growth [18, 20], climate change [23, 24], and policy intervention [20] are the main motivations for promoting change in the apple production layout of China. Third, some researchers, such as Zhang et al. [25] and Feng and Huo [26], analysed the spatial agglomeration characteristics of the apple environment total factor productivity (ETFP) and found that the development of the apple ETFP in the main apple production areas in China had obvious spatial correlation. Wang and Wei [27] believed that the trend of changes in the spatial layout of apple production and seasonal changes are the main reasons for apple price fluctuations.

In summary, first, most studies focused on the time series changes, geographical aggregation characteristics, and influencing factors of crops, including grain [28, 29], vegetables [30], tea [18, 20], and peanut [31]. However, the understanding of the spatial pattern of China’s apple production is still very limited. Although some researchers have used different research methods to analyse the characteristics of regional pattern changes in China’s apple production over a certain period from different spatial scales, the research conclusions are quite different, due to the different research time intervals, methodological approaches, and measurement indicators, and it is difficult to fully reflect the evolution of the production pattern and the characteristics of agglomeration since the marketization reform of China’s apple industry in 1978. Second, due to the wide and diverse nature of apple production areas in China, the significant differences in regional resource endowments, technical equipment levels, market development, supply-demand relationship, and government industrial support policies, the production layout, and the spatial development of apples are uneven and uncoordinated. The geographical pattern of production fluctuates greatly. Since the reform and opening up, certain questions have arisen, such as, what kind of spatial-temporal dynamics characteristics exist in China’s apple-producing areas? What are the factors that affect the apple production layout? The existing literatures cannot provide an in-depth analysis of these issues and ignore the impact of spatial effects on the apple production layout. Third, under the realistic background of the significant change in the relationship between production supply and market demand, it is important to ensure the supply stability and regional balance of apple supply and demand and to adjust apple industrial policies and regional layout in the process of promoting agricultural supply-side structural reform and implementing a rural revitalization strategy. Therefore, to fill the existing gap, and to provide a scientific basis for optimizing and adjusting apple production layout and institutional arrangements, stabilizing and improving China’s apple production level, this study revealed the dynamic evolution trend and the spatial-temporal characteristics of China’s apple production layout from the national and provincial perspectives. Moreover, this study used geographic analysis methods and spatial econometric models to incorporate spatial interaction effects into empirical analysis to explore the driving factors affecting the apple production layout.

Thus, the rest of this study is structured as follows: Section 2 theoretically discusses the influencing factors of apple production layout. Section 3 introduces the data and primary methods used in the analysis. Section 4 provides a descriptive analysis of spatial-temporal dynamics of apple production in China. Section 5 uses a spatial econometric model to empirically analyse the factors affecting the apple production layout. Section 6 concludes with a summary of the major findings and their policy implications.

## Theoretical analysis

According to the theory of agricultural production economics, apple production is an organic combination of natural and economic reproduction. In the process of natural reproduction, apple production is bound by natural resources, such as climate and land resources [32]. In the process of economic reproduction, apple production is subject to levels of economic and social development, the comparative benefit of crops, scientific and technological progress, infrastructure development, and policy interventions. The combined effects of the above factors have motivated the apple production layout. Therefore, based on the six factors of resource environment, opportunity cost, infrastructure, technological progress, consumer demand, and policy environment, this study combined the actual situation of China’s apple production development and data accessibility and empirically analysed the influencing factors of China’s apple production layout. Referring to existing research, this study used the apple area concentration index (i.e. the proportion of apple area in each province to the total apple area nationwide) to characterize the agglomeration degree of apple production layout.

### Resource environment

Natural disasters caused by changes in climate factors, such as temperature and rainfall, directly affect apple yield and quality [18, 28], which in turn affect apple production efficiency and farmers’ enthusiasm. China’s apple production areas are widely distributed, and the climatic conditions vary greatly among provinces [33]. The natural disasters and the degree of disasters caused by climate change affect the layout of apple production [29, 32]. This study used the area of apple production affected by disasters to characterize the impact of natural disasters on apple production in each province. In addition, according to the resource endowment theory, regions should produce and export products based on their relatively abundant factors. As one of the most basic agricultural production resources, cultivated land resources are the main resource constraint affecting apple production. Generally, there is a positive relationship between the area of apple production and cultivated land resources, but with the advancement of industrialization and urbanization, the reduction of cultivated land resources and population growth have led to a decrease in per capita arable land area [34]. The changes in per capita arable land area in different provinces and regions also vary due to differences in industrialization and urbanization development levels and population changes. This study used the per capita arable land area of each province to characterize their status of cultivated land resources. Based on the above analysis, the following hypotheses were made:

H1: The disaster area of apples has a negative impact on the agglomeration of apple production layout.

H2: Per capita arable land area has a positive impact on the agglomeration of apple production layout.

### Opportunity costs

According to the theory of comparative advantage and the assumption of the “rational economic man”, farmers choose whether to engage in agricultural production based on the principle of maximizing income [35]. Due to the uncertainty of output caused by the natural and market risks of agricultural production, the lack of comparative advantages of agriculture over non-agricultural industries, coupled with the rise in labour wages of non-agricultural industries, has led to an increase in the opportunity cost of apple cultivation. Therefore, the thrust formed by the inherent weakness of agriculture and the pull of the high remuneration in non-agricultural industries had jointly promoted the shift of agricultural labour to non-agricultural industries [36]. The differences in the development level of non-agricultural industries in different provinces and regions led to the creation of different non-agricultural employment opportunities for apple farmers, thus, provinces and regions with high non-agricultural opportunities may have less apple production areas and vice versa. This study used the proportion of rural non-agricultural labour to rural labour in each province to characterize non-agricultural employment opportunities in each province. Moreover, besides the choice between agricultural and non-agricultural industries, farmers also need to choose a planting structure within the agricultural system. If income from apple production is higher than that of other crops, farmers are more inclined to grow apples. Therefore, the comparative benefit of apple production will also have an impact on the area of apple production [30]. Due to the lack of data on the net profit per acre of apples and other crops in some provinces, this study employed the methods used by Zhang and Wang [31], where the proportion of area of the apple production to a total area of crop production in each province was used to characterize the comparative benefits of apple production in the corresponding province. Based on the above analysis, the following hypotheses were made:

H3: Non-agricultural employment opportunities have a negative impact on the agglomeration of apple production layout.

H4: Comparative apple income has a positive impact on the agglomeration of apple production layout.

### Infrastructure

Apple cultivation is a natural reproduction process, and its growth requires sufficient water. However, most of China’s apple production areas are distributed in the northern mountainous and hilly areas, where the climate is mainly dry and less rainy [5]. Therefore, the conditions of irrigation facilities are important factors affecting apple cultivation [28]. This study used the effective irrigated area of apples in each province to characterize the conditions of their apple irrigation facilities. Moreover, according to the agricultural location theory, the geographic distance of agricultural production and sales determines the land use structure. As a high-value agricultural product (HVP), apple is highly commoditized and marketized. The transportation conditions are the link connecting apple production to consumption and are also an important condition for realizing apple market value [18]. Therefore, the transportation conditions in each province affect its apple production area. The land transport density of each province is used to characterize its transportation conditions. Based on the above analysis, the following hypotheses were made:

H5: Apple irrigation facilities have a positive impact on the agglomeration of apple production layout.

H6: Traffic conditions have a positive impact on the agglomeration of apple production layout.

### Technological progress

The impact of technological progress on apple production layout is mainly reflected in the apple yield level. The progress of apple production technology is conducive to the improvement of apple production efficiency and economic benefits [18]. However, due to the differences in the levels of production technology innovation and promotion in various provinces, the apple’s comparative income with other crops has changed, which in turn affects the apple production layout. Most scholars used time trends to express technological advancement variables, but for apple production, technological progress is more manifested in yield increase. Therefore, this study used apple yield rather than a time trend to indicate technological advancement. Considering 1978 as a base year, the apple yield was set to be 1 in 1978, and the standardized yield from 1979–2016 is used to determine the technological advancement of apples in each province. Based on the above analysis, the following hypothesis was made:

H7: The apple’s technological advancement has a positive impact on the agglomeration of apple production layout.

### Consumption demand

According to the agricultural location theory, the high transportation costs and product losses of long-distance transportation have limited agricultural production and circulation to a certain area. Due to the high degree of commercialization, the demand for apple consumption stimulates the growth of apple production in the region. In general, apple consumption demand is positively related to the size and density of the population [17]. This study used the proportion of the total population at the end of the year in each province to the total national population at the end of the year to characterize the potential apple consumption demand in each province. Based on the above analysis, the following hypothesis was made:

H8: Apple consumption demand has a positive impact on the agglomeration of apple production layout.

### Policy environment

Changes in national policies have an impact on the enthusiasm of apple farmers and apple production area of the region [37]. For example, to ensure national food security, the government imposed the “*Provincial Governor Responsibility System*” (PGRS) policy from 1995 to 1997 [38, 39]. However, due to limited arable land resources, this policy has a certain crowding-out effect on apple production. In 2003 and 2009, the government released the *Apple Advantage Regional Development Plan (2003–2007)* and *Apple Advantage Regional Planning (2008–2015)* (AARP), and the implementation of the advantageous regional layout plans has a certain impact on the agglomeration of apple production layout [18, 40]. Based on the above analysis, the following hypotheses were made:

H9: The PGRS has a negative impact on the agglomeration of apple production layout.

H10: The AARP has a positive impact on the agglomeration of apple production layout.

## Data sources and methodology

### Data sources and description

In the absence of farm-level panel data, aggregate data are often used for analysing the factors influencing China’s apple production layout. Evaluations relying on aggregate data can reveal the characteristics of China’s apple production layout agglomeration at the regional or national levels. The data set used in the present study covered 23 apple-producing provinces in China’s mainland for thirty-nine consecutive years (1978–2016), with a total of 858 observations. To maintain data consistency and comparability, this study incorporated Chongqing data into Sichuan province. In this way, there are 22 provinces. Data used in this study were derived from the *China Rural Statistical Yearbook (1985–2018)*, *Statistics of Agriculture in New China for 60 Years*, *China Statistical Yearbook (1981–2018)*, and statistical yearbooks of various provinces.

According to *the Apple Regionalization Plan* of China Agriculture Research System (CARS), China’s apple production area is divided into five regions, including the Bohai Bay production area (including Shandong, Hebei, Liaoning, Tianjin, and Beijing), the Loess Plateau production area (including Shaanxi, Gansu, Shanxi, Ningxia, and Qinghai), the Yellow River Old Road production area (including Henan, Anhui, and Jiangsu), the Southwest Cold and Highland production area (including Sichuan, Chongqing, Yunnan, Guizhou, and Tibet), and the Special production area (including Inner Mongolia, Heilongjiang, Jilin, Hubei, and Xinjiang). Among them, the Bohai Bay and the Loess Plateau production areas are the advantageous apple-producing areas in China [5].

### Empirical methodology

#### Apple production concentration index (*APCI*)

This study used the apple production concentration index to analyse the evolution characteristics of regional apple production patterns in China. This indicator not only comprehensively examines the contribution of apple output in each province to the total national apple output in a certain period of time but also compares differences in the growth rate of apple output in each province. The calculation formula is as follows:

$$C_{i}=\frac{P_{i}}{\sum_{i=1}^{22}P_{i}}$$ (1)

where *C*_*i*_ and *P*_*i*_ indicate the apple production concentration index (*APCI*) and apple output of the provinces *i*, respectively.

#### Exploratory spatial data analysis (ESDA)

Spatial autocorrelation is a crucial method of ESDA [41, 42]. It reveals the interaction mechanism between spatial agglomeration and spatial heterogeneity between research objects through the description and visualization of certain geographical phenomena [43] and is a measure of the agglomeration degree of a certain geographical phenomenon in the spatial domain [44, 45]. In this study, spatial autocorrelation is used to test whether the area of apple production in a certain province is significantly correlated with the areas of apple production in neighbouring provinces. According to the spatial autocorrelation coefficient, it is divided into positive and negative correlations. A positive correlation indicates that the area of apple production in a certain province has the same trend as the areas of apple production in its neighbouring provinces, while a negative correlation indicates just the opposite. According to the size of the spatial analysis range, spatial autocorrelation can be divided into global and local spatial autocorrelation.

Global spatial autocorrelation is used to describe the spatial characteristics of apple production nationwide and to determine whether there are agglomeration characteristics in apple production in space. Spatial statistics use the global spatial autocorrelation statistics, such as *Global Moral’s I*, *Global Geary’s*, and *Gettis’ G*, to measure the spatial correlation and spatial difference of the overall region. Compared with other global spatial autocorrelation statistics, *Global Moral’s I* index is less susceptible to deviation from normal distribution [46, 47]. Therefore, this study chooses *Global Moral’s I* index to measure the global spatial autocorrelation of China’s apple production. The specific calculation method is as follows:

$$Global\;Moran\prime s\;I=\frac{1}{\sum_{i=1}^{n}\sum_{j=1}^{n}w_{ij}}*\frac{\sum_{i=1}^{n}\sum_{j=1}^{n}w_{ij}(x_{i}-\bar{x})(x_{j}-\bar{x})}{{\sum_{i=1}^{n}\left(x_{i}-\bar{x}\right)}^{2}/n}$$ (2)

where *x*_*i*_ and *x*_*j*_ indicate the apple areas of the provinces *i* and *j*, respectively, *i*, *j* = 1, 2, ⋯, *n* (*n* is the total number of provinces, *n* = 22), $\bar{x}$ is the average area of apple production in all provinces, and *w*_*ij*_ indicates the elements of the spatial weight matrix *W*. The value of *Global Moral’s I* index is between -1 and 1. A positive value of *Global Moral’s I* indicates a positive spatial autocorrelation in the spatial distribution of apple area in neighbouring provinces and a negative value indicates a negative spatial autocorrelation. The closer the value of *Global Moral’s I* is to ±1, the stronger the space autocorrelation of apple production is; the value of 0 indicates that there is no spatial autocorrelation in apple production and it is spatially random.

Global spatial autocorrelation focuses on analysing the spatial distribution of a certain geographical phenomenon in the overall regional space, ignoring the spatial correlation of this geographical phenomenon in some local areas, and thus local spatial autocorrelation is used to analyse whether there is high- or low-value aggregation in apple production between each province and its neighbouring provinces. This study used the local spatial autocorrelation index (*Local Moral’s I* index) and the spatial association local indicator Local Indicators of Spatial Association (LISA) to measure the spatial agglomeration, heterogeneity, or random distribution characteristics of apple production in various local areas [48]. The specific calculation method is as follows:

$$Local\;Moral\prime s\;I=\frac{n(x_{i}-\bar{x})\sum_{j=1}^{n}w_{ij}(x_{j}-\bar{x})}{\sum_{i=1}^{n}{\left(x_{i}-\bar{x}\right)}^{2}}$$ (3)

where *n*, *x*_*i*_, *x*_*j*_, $\bar{x}$, *w*_*ij*_ have the same meaning as in Eq 2. The *Local Moral’s I* index has a similar meaning to the *Global Moral’s I* index. A positive *Local Moral’s I* indicates that the high (low) value of a province is surrounded by high (low) values, and a negative *Local Moral’s I* indicates that the high (low) value of a province is surrounded by low (high) values.

Local spatial autocorrelation can visualize the spatial pattern of the local differences in apple production through Moran scatter plots and local spatial autocorrelation clustering maps. Moran scatter plots and local spatial autocorrelation cluster plots are based on the numerical magnitude of *Local Moral’s I* and its significance, and the local spatial units are divided into HH (high-high), LH (low-high), LL (low-low), HL(high-low), and other types [49], where the HH indicates that the apple area of a certain province and its neighbouring provinces is relatively large; the LH suggests that the apple area of a certain province is small, but the area of its neighbouring provinces is larger; the LL indicates that the apple area of a province and its neighbouring provinces are relatively small; and the HL indicates that the apple area in a certain province is larger, but that of its neighbouring provinces is smaller. The left spatially randomly distributed regions indicate that *Local Moral’s I* did not pass the significance test and that the spatial agglomeration was not strong.

#### Spatial econometric model

In some areas, the relatively high price spill-over effect of apple induced the expansion of the production area in adjacent areas, which led to the spatial correlation of apple production layout. However, the existing research on the layout of apple production mostly used panel data models in the empirical analysis, which masks the role of spatial differences and ignores the influence of spatial effects on the agglomeration of apple production layout. At present, the spatial econometric analysis method is an effective means to quantitatively study the layout of agricultural production. The spatial econometric analysis method abandons the assumption of the traditional econometric that space is homogeneous and adds spatial interaction effects to the econometric model, which can provide more accurate analysis. The spatial correlation of the subject is the premise and basis for constructing the spatial econometric model. Therefore, based on the spatial correlation analysis, this study further used the spatial panel data model to quantitatively analyse the influencing factors of China’s apple production layout agglomeration. The spatial panel data models mainly include the Spatial Lag Regression (SLR) model, the Spatial Error Model (SEM) and the Spatial Dubin Model (SDM). Among them, the SDM is the general form of the SLR and SEM models and is also a standard framework for capturing various types of spatial spill-over effects [44]. Therefore, this study used the SDM for empirical analysis. The matrix form of the model is as follows:

$$R_{it}=\lambda WR_{it}+\beta X_{it}+\delta WX_{it}+\varepsilon$$ (4)

where *R* indicates the concentration index of the apple area, *W* indicates the adjacent spatial weight matrix or the geographic distance spatial weight matrix, *WR* indicates the spatial lag term of the apple area concentration index, and *X* indicates the influencing factors of China’s apple production layout agglomeration. *ε* reports the error term.

## The spatial-temporal dynamics of apple production

### Temporal dynamics of apple production

**Fig 1:**
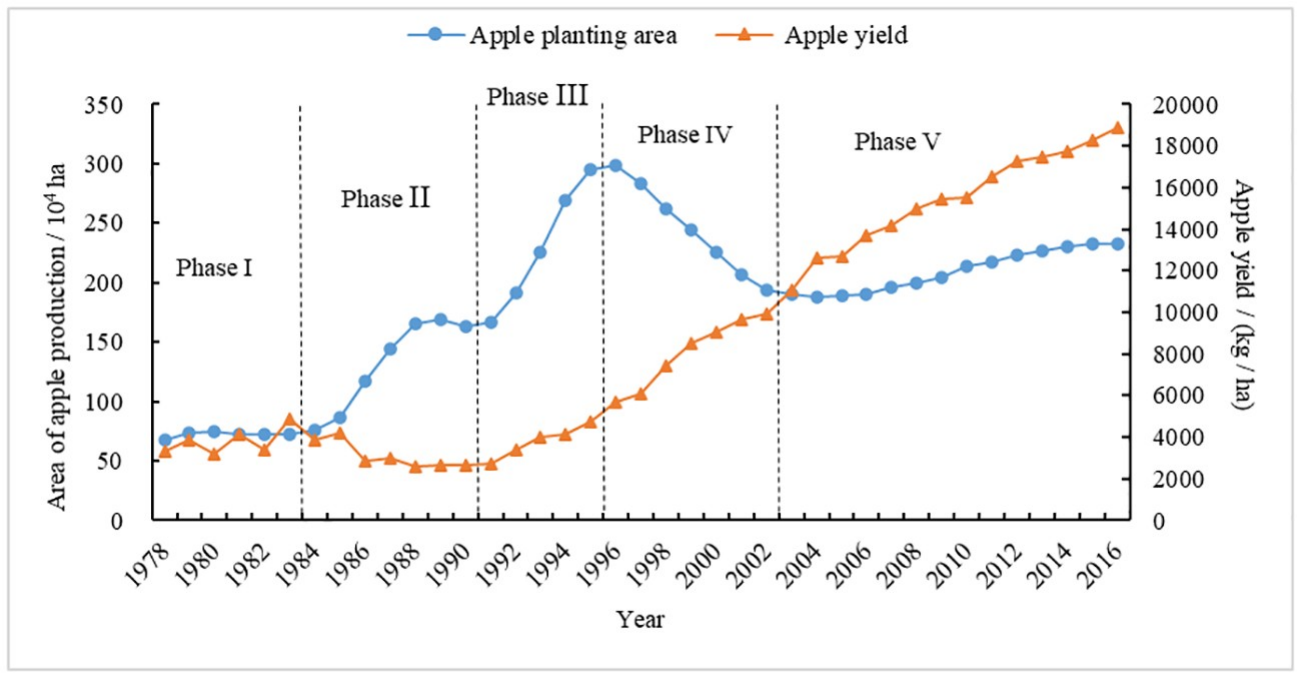
Changes in apple planting area and yield in China, 1978–2016. *Note*: The division of the apple production stage is based on the changes in apple planting area. *Source*: China Rural Statistical Yearbooks [2].

**Fig 2:**
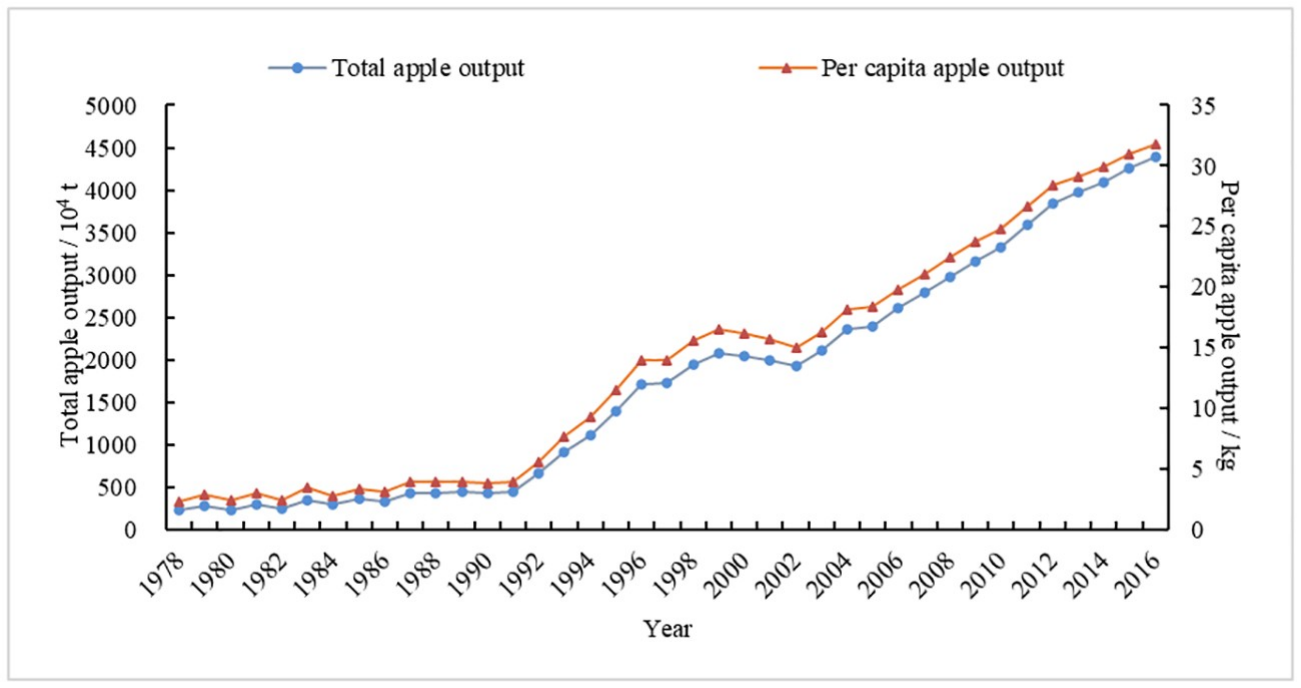
Changes in total apple output and per capita output in China, 1978–2016. *Source*: China Rural Statistical Yearbooks and China Statistical Yearbooks [2, 3].

From the analysis of changes in apple production in China (Figs 1 and 2), its volatility is obvious. The area of apple plantation, total output, per capita output, and yield have shown a volatile growth trend. Among them, the fluctuation of plantation areas is larger. From its changes, apple production in China has gone through the following five stages:

1. Phase I (Stable period (1978–1984)): apple production areas remained essentially unchanged, maintained at approximately 0.70 million ha, and the total output, yield and per capita output of apples were also stable. The reason is that although the rural economic system reform began in 1978 and the rural commodity economy was active, it was not compatible with production development and social needs due to the single distribution channels of fruits, the restrictions on purchase and sale policies, price management, and the weak construction of circulation infrastructure. The contradiction was prominent, which led to the slow development of China’s apple production and sales. From 1978 to 1984, China’s fruit business implemented a planning management model with the supply and marketing cooperative as the main body, which restricted the development of apple production.
2. Phase II (Rapid growth period A (1985–1991)): the area under apple production expanded rapidly, from 0.87 million ha in 1985 to 1.66 million ha in 1991, with an average annual growth rate of 11.48 percent. The total apple output and per capita output remained basically unchanged, while apple yield declined. The findings revealed that, although the area of apple production rapidly expanded during this period, the apple production system still used the traditional cultivation methods and techniques, which might be the main reason for the declined apple yield. Therefore, the effect of increasing the area of apple production was not obvious.
3. Phase III (Rapid growth period B (1992–1996)): the area of apple production increased sharply from 1.91 million ha in 1992 to 2.99 million ha in 1996, with an average annual growth rate of 11.76 percent. In response, the total output, yield, and per capita output increased rapidly. In particular, the national apple area reached a record high level in 1996, with the total apple output close to 0.14 million tons, and the apple yield increased from 3,424.39 kg/ha in 1992 to 5,707.45 kg/ha in 1996, with an average annual growth rate of 13.62 percent. The results revealed that, although the apple yield has increased yearly due to the introduction of new varieties and the improvement of production technology, the contribution of the increased area of apple production was still greater than the corresponding yield. It can be seen that the increase in apple production during this period still depended on the traditional method of increasing production area.
4. Phase IV (Rapid decline period (1997–2003)): with an average annual growth rate of -6.47 percent, the apple production area rapidly decreased from 2.84 million ha in 1997 to 1.90 million ha in 2003. The apple yield increased from 6,066.66 kg/ha to 11,103.98 kg/ha, with an average annual growth rate of 10.60 percent, which offset the impact of production area reduction on total apple output. The reason is that since 1997, national apple production has entered a stage of adjustment, which has greatly reduced the low-effect apple orchards, such as unsuitable varieties, old orchards, and poorly managed orchards in both the unsuitable and suitable areas for apples, while the apple area in the apple eugenic and high-economy areas developed steadily. At the same time, the apple yield level has increased rapidly, and its effect on increasing output has become increasingly visible due to several factors such as the use of improved varieties, technology innovation, upgrading of orchard equipment and infrastructure, and improving the skills of fruit farmers.
5. Phase V (Slow growth period (2004–2016)): though the apple area increased gradually from 1.88 million ha in 2004 to 2.32 million ha in 2016, with an average annual growth rate of 1.80 percent, the apple yield increased from 12,615.90 kg/ha to 18,883.72 kg/ha, resulting in a continuous increase in total apple output and per capita output. The reason is that since 2001, China’s apple production has gradually changed from quantitative to quality-effective, the apple production area has become more reasonable, and production has entered into a new stage of industrialization. Meanwhile, with the continuous improvements in apple production technology and management level since 2003, the contribution of yield in total apple output has continued to be higher than the increase in output due to the increase of the apple production area.

In general, China’s apple area, total output, per capita output, and yield all increased from 1978 to 2016 to different degrees. Among them, the area of production increased from 0.68 million ha to 2.32 million ha, with an average annual growth rate of 3.29 percent. The total apple output increased from 2.28 million tons to 43.88 million tons, with an average annual growth rate of 8.10 percent. Likewise, per capita apple output increased from 2.36 kg to 31.74 kg, with an average annual growth rate of 7.08 percent. Similarly, with an average annual growth rate of 4.66 percent, the apple yield increased from 3,351.17 kg/ha to 18,883.72 kg/ha.

### Spatial concentration characteristics of apple production

1. As the concentration of apple production in the advantage area of Bohai Bay continued to decline, the concentration of apple production in the Bohai Bay area dropped from 75.57 percent in 1978 to 36.76 percent in 2016, with a drop of 38.81 percent (Table 1). In this area, the concentrations of apple production in Shandong and Liaoning, which are the main producing areas of the Bohai Bay, decreased significantly (15.16 percent in Shandong and 22.31 percent in Liaoning), resulting in an overall decline in the concentration of apple production in the Bohai Bay area. Since 2010, the Bohai Bay area has dropped from China’s largest apple-producing area to the second largest. In addition, the concentrations of apple production in Beijing and Tianjin decreased by 1.94 percent and 0.16 percent, respectively, while the concentration of apple production in Hebei increased by 0.76 percent.
2. The importance of apple production in the dominant area of the Loess Plateau is prominent. The concentration of apple production in the dominant area of the Loess Plateau increased from 10.63 percent in 1978 to 44.37 percent in 2016, an increase of 33.74 percent, making it the largest apple-producing area in China. In this area, Shaanxi has the best performance, with its apple production concentration increasing from 4.36 percent in 1978 to 25.08 percent in 2016, an increase of 20.72 percent. Since 2009, Shaanxi has surpassed Shandong to become the largest apple production province in China. In addition, the concentrations of apple production in Gansu, Shanxi, and Ningxia increased by 6.32 percent, 5.91 percent, and 0.85 percent, respectively (Table 1).
3. Apple production in the Yellow River Old Road production area continued to be stable. The concentration of apple production in the Yellow River Old Road production area increased slightly, from 8.55 percent in 1978 to 12.13 percent in 2016, with an increase of 3.58 percent. In this area, the concentration of apple production in Henan, which is the main producing area of the Yellow River Old Road production area, increased by 3.09 percent. Moreover, the concentrations of apple production in Anhui and Jiangsu increased by 0.36 percent and 0.13 percent, respectively.
4. The status of apple production in the Southwest Cold and Highland production area remained essentially unchanged, with its apple production concentration merely increasing by 0.20 percent from 1978 to 2016. In this area, the concentration of apple production in Yunnan and Guizhou increased by 0.41 percent and 0.07 percent, respectively, while the concentration of apple production in Tibet decreased by 0.10 percent.
5. The concentration of apple production in the Special production area increased slightly. The concentration of apple production in the Special production area increased from 2.88 percent in 1978 to 4.19 percent in 2016, with an increase of 1.31 percent. Xinjiang is a typical representative in the Special production area, and its apple production concentration increased from 1.78 percent in 1978 to 3.11 percent in 2016, showing an increase of 1.33 percent. Additionally, the concentration of apple production in Heilongjiang and Inner Mongolia remained mostly unchanged, while the concentration of apple production in Jilin and Hubei decreased by 0.32 percent and 0.09 percent, respectively (Table 1).

**Table 1:** Changes in apple production concentrations in provinces and regions, 1978–2016 (percent).

Apple division	Province	Year								
		1978	1980	1985	1990	1995	2000	2005	2010	2016
**Bohai Bay production area**	Shandong	37.45	38.86	40.23	33.15	35.87	31.70	27.97	24.02	22.29
	Hebei	7.57	7.53	12.95	10.84	8.97	8.84	9.17	8.19	8.33
	Liaoning	28.15	25.82	15.16	17.57	9.12	6.03	5.41	6.30	5.85
	Tianjin	0.29	0.35	0.64	0.72	0.45	0.39	0.28	0.17	0.12
	Beijing	2.11	1.93	1.41	1.71	0.95	0.77	0.58	0.31	0.17
	Subtotal^a^	75.57	74.50	70.39	63.99	55.36	47.73	43.41	38.98	36.76
**Loess Plateau production area**	Shaanxi	4.36	3.78	3.90	8.08	16.69	19.02	23.33	25.73	25.08
	Gansu	1.89	2.51	2.71	4.05	3.23	3.38	4.22	6.06	8.21
	Shanxi	3.86	3.66	4.79	3.38	4.96	7.98	6.87	7.72	9.77
	Ningxia	0.45	0.55	0.75	0.81	0.66	0.78	0.93	1.07	1.30
	Qinghai	0.08	0.18	0.33	0.32	0.12	0.07	0.03	0.02	0.01
	Subtotal^a^	10.63	10.68	12.48	16.65	25.66	31.22	35.37	40.60	44.37
**Yellow River Old Road production area**	Henan	6.91	6.55	7.69	8.29	11.09	11.69	12.52	12.29	9.99
	Anhui	0.49	0.76	0.72	1.34	1.21	1.48	1.16	1.22	0.85
	Jiangsu	1.16	2.05	1.83	2.45	2.30	3.40	2.30	1.70	1.29
	Subtotal^a^	8.55	9.36	10.24	12.09	14.59	16.57	15.98	15.22	12.13
**Southwest Cold and Highland production area**	Sichuan	1.62	1.80	1.33	1.46	0.90	1.02	1.04	1.31	1.44
	Yunnan	0.55	0.47	0.53	0.72	0.45	0.49	0.66	0.78	0.96
	Guizhou	0.07	0.06	0.17	0.09	0.04	0.04	0.04	0.05	0.14
	Tibet	0.12	0.14	0.11	0.09	0.00	0.03	0.02	0.02	0.01
	Subtotal^a^	2.35	2.46	2.13	2.36	1.39	1.58	1.77	2.14	2.55
**Special production area**	Inner Mongolia	0.23	0.40	0.55	0.53	0.26	0.23	0.26	0.23	0.40
	Jilin	0.63	0.62	0.17	0.25	0.28	0.49	1.05	0.46	0.31
	Heilongjiang	0.12	0.14	0.44	0.53	0.54	0.55	0.74	0.35	0.34
	Hubei	0.12	0.13	0.19	0.28	0.23	0.15	0.05	0.03	0.03
	Xinjiang	1.78	1.59	3.46	3.26	1.69	1.47	1.38	1.98	3.11
	Subtotal^a^	2.88	2.88	4.81	4.86	3.00	2.88	3.48	3.06	4.19

^a^ The “subtotal” in the table is the concentration of apple production in each production area.

In summary, the concentration of apple production in the dominant area of the Loess Plateau has continued to increase and has become China’s largest apple production area. The Bohai Bay area has gradually declined to become the second-largest apple production area in China. From the perspective of the distribution pattern of apple production provinces, the top 6 provinces in apple production concentration were transformed from Shandong, Liaoning, Hebei, Henan, Shaanxi, and Shanxi in 1978 to Shaanxi, Shandong, Henan, Shanxi, Hebei and Gansu in 2016. The concentrations of apple production in some provinces in the central and western regions (such as Shaanxi, Shanxi, and Gansu) continued to rise, and these regions have gradually become China’s main apple production provinces. The reason is that China’s apple production technology is relatively backward overall at present, as most apple production links, such as fertilization, pesticide application, bagging, pruning and fruit picking, were mainly completed by laborers. With the rapid progress of industrialization and urbanization in the Bohai Bay area, a large number of high-quality labourers were off-farm, resulting in the rising labour costs of apple production, which in turn prompted the transfer of apple production from the expensive labour areas in the east to the inexpensive labour areas in the central and western regions. It can be seen from the production area and the provincial level that the centre of China’s apple production is moving southwest, shifting from the dominant area of the Bohai Bay to the dominant area of the Loess Plateau.

### Spatial autocorrelation of apple production

GeoDa v.1.12 and ArcGIS10.3.1 software were used to carry out the spatial autocorrelation test of apple areas in various provinces of China from 1978 to 2016. The results are shown in Table 2.

**Table 2:** The *Global Moral’s I* index of apple area, 1978–2016.

Year	*Moral’s I*	*Z*	Year	*Moral’s I*	*Z*	Year	*Moral’s I*	*Z*
**1978**	0.2079**	2.3847	1991	0.2238**	2.4599	2004	0.2128**	2.2665
**1979**	0.2050**	2.3528	1992	0.1992**	2.3689	2005	0.2060**	2.2128
**1980**	0.2112**	2.3864	1993	0.2290**	2.5370	2006	0.1903**	2.1071
**1981**	0.2130**	2.3836	1994	0.2659***	2.7755	2007	0.1853**	2.0631
**1982**	0.2143**	2.4002	1995	0.2679***	2.7796	2008	0.1766**	2.0624
**1983**	0.2158**	2.4306	1996	0.2646***	2.7438	2009	0.1653**	1.9952
**1984**	0.2131**	2.4180	1997	0.2588***	2.6857	2010	0.1550*	1.9171
**1985**	0.2343***	2.6751	1998	0.2549***	2.6325	2011	0.1521*	1.9160
**1986**	0.2558***	2.8847	1999	0.2593***	2.6485	2012	0.1449*	1.8581
**1987**	0.2608***	2.9024	2000	0.2522***	2.5794	2013	0.1397*	1.8015
**1988**	0.2697***	2.9517	2001	0.2442**	2.5070	2014	0.1340*	1.7552
**1989**	0.2661***	2.8881	2002	0.2337**	2.4206	2015	0.1299*	1.7308
**1990**	0.2287**	2.5408	2003	0.2178**	2.3025	2016	0.1194	1.6416

***, **, and * represent significance level at 1%, 5%, and 10%, respectively.

The *Global Moral’s I* index of the apple area in each province from 1978 to 2016 is calculated using Eq 2. The results are shown in Table 2. It can be seen that the *Global Moral’s I* index of apple area has been positive over the years, and most of the *Global Moral’s I* index has passed at least the 10 percent significance level test, indicating that apple production in China at the provincial level has not been randomly distributed in space since 1978 and has significant spatial autocorrelation. The areas with relatively close apple production levels showed spatial agglomeration characteristics. That is, if a province has a large apple area, so do their neighbouring provinces. Similarly, if a province has a smaller apple area, so do their neighbouring provinces. China’s apple production presented a significant spatial spill-over effect.

Over time, the *Global Moral’s I* index of apple acreage showed an “M-type” change trend, and the *Z* value of the test statistic showed the same trend. Overall, the *Global Moral’s I* index and *Z* statistic of the apple area showed a downward trend, which decreased from 0.2079 and 2.3847 in 1978 to 0.1194 and 1.6416 in 2016, indicating that the spatial agglomeration effect of China’s apple production has been wave-like since 1978. As time progresses, the spatial agglomeration degree of the apple area is gradually decreasing.

Using Eq 3, the *Local Moral’s I* index value of the apple area in each province of China is calculated using the GeoDa0.95i and ArcGIS10.2 software, and the areas where the *Local Moral’s I* index passes the 5 percent significance level test were divided into HH (high-high), LH (low-high), LL (low-low) and HL (high-low) types. The result showed that the number of HH and LL types decreased from 7 provinces in 1978 to 6 provinces in 2016, indicating that the spatial agglomeration characteristics of apple area were weakened, and this result is consistent with the result of the global space autocorrelation analysis of the apple area. Specifically, the regions with smaller apple areas are mainly distributed in the east, central, and southern China regions, which are south of the Yangtze River. The regions with large apple areas are mainly distributed in the central part of the north. The areas of production in HH-type provinces (Hebei and Henan) in 1978 were transformed to Shanxi and Henan provinces in 2016. The high agglomeration apple area moved to the southwest. In 2016, the spatial correlations of the six provinces (including Shanxi, Henan, Hunan, Jiangxi, Fujian, and Guangdong) were all tested by the 5 percent significance level. Among them, Shanxi and Henan provinces and their neighbouring provinces had more areas of apple production, and they belonged to the hot spot area. The areas of apple production in Hunan, Jiangxi, Fujian, and Guangdong provinces and their neighbouring provinces were relatively small, belonging to the cold spot area. This shows that China’s apple production is mainly concentrated in the central part of the north, centred on Shanxi and Henan.

### The influencing factors of spatial-temporal dynamics of apple production in China

#### Explanatory variables

According to the theoretical analysis, from the six aspects of resource environment, opportunity costs, infrastructure, technological progress, consumption demand, and policy environment, we chose variables such as apple disaster areas, per capita arable land, non-agricultural employment opportunities, apple comparative income, apple effective irrigation area, transportation density, apple yield, population proportion, food policy and industry planning to explain the spatial-temporal dynamics of China’s apple production. Table 3 presents the meaning and summary statistics of the variables.

**Table 3:** Variable settings and meaning.

Variables		Variable meaning	Mean values	S.D
**Dependent variable**	The concentration index of apple area (percent)	The proportion of apple area in each province to the total area of apple production in the nationwide	0.0454	0.0648
**Resource environment**	Apple disaster areas (10^3^ha)	Crop disaster area * (apple area / total crop production area)	26.8401	44.9563
	Per capita arable land (10^3^ha/ 10^4^ persons)	Cultivated land area / rural labor	5.7842	4.4804
**Opportunity costs**	Non-agricultural employment opportunities (percent)	(Rural labor—agriculture, forestry, animal husbandry and fishery practitioners) / rural labor	0.2712	0.1778
	apple comparative income (percent)	Apple area / total crop production area	0.0174	0.0239
**Infrastructure**	Apple effective irrigation area (10^3^ha)	Crop effective irrigated area * (apple area / total crop area)	30.8026	47.7399
	Transportation density (km/10^4^ km^2^)	(Railway + highway) / administrative area	3949.3961	4008.0512
**Technological progress**	Apple yield (kg/ha)	Apple output / apple area	6935.8932	6301.6315
**Consumption demand**	The proportion of population (percent)	Provincial population / national population	0.0329	0.0240
**Policy environment**	PGRS	1995–1997 are 1 and the remaining years are 0	0.0769	0.2666
	AARP	2003–2015 are 1 and the remaining years are 0	0.3333	0.4717

### Estimation results and discussion

Based on *Moral’s I* index, there was significant spatial autocorrelation in apple production. If spatial autocorrelation is ignored, the regression analysis may exhibit a “pseudo-regression” phenomenon. To select the most suitable model, this study used the Stata version 15.0 software package for the likelihood ratio (LR) and Hausman tests. The p-value of the LR test is 0.0000, so we can reject the null hypothesis that the SDM can degenerate into the SLR or the SEM. The Hausman statistic is -1,039.36, which is negative, so the null hypothesis of the random effect can be accepted. Therefore, it is most appropriate to finally determine the SDM with random effects using Stata version 15.0 software. The SDM model estimation results are shown in Table 4.

**Table 4:** The estimation results of the space Dubin Model.

Explanatory variable	Adjacency matrix		Geographic distance matrix	
	Coefficient	Robust standard error	Coefficient	Robust standard error
**Apple disaster areas**	0.0343***	0.0129	0.0319**	0.0135
**Per capita arable land**	0.0771	0.0682	0.0350	0.0653
**Non-agricultural employment opportunities**	-0.0293	0.0376	-0.0551	0.0373
**Comparative apple income**	0.5225***	0.1270	0.4835***	0.1186
**Apple effective irrigation area**	0.3983***	0.1249	0.4371***	0.1139
**Transportation density**	0.2016***	0.0696	0.1962**	0.0774
**Apple yield**	0.0170	0.0210	0.0174	0.0171
**The proportion of the population**	0.3318	0.3015	0.2506	0.2603
**PGRS**	-0.0408***	0.0391	-0.1170***	0.0120
**AARP**	0.0848***	0.0480	0.0677***	0.0094
**Constant term**	-2.0149	1.4936	-1.9687	1.6976
**Observed number**	858		858	
*R*^2^	0.8913		0.9273	

***, **, and * represent significance level at 1%, 5%, and 10%, respectively.

As illustrated in Table 4, the SDM using two spatial weight matrices has consistent estimation results, indicating that the model estimation results were relatively robust. Since the SDM using the geographic weight matrix had a better goodness-of-fit (*R*^2^ = 0.9273), the interpretation of the estimation results is based on the estimation results of the model. From the significance of each coefficient, comparative apple income, apple effective irrigated area, transportation density, the PGRS, the AARP, and apple disaster area have significant effects on the agglomeration of the apple production layout with decreasing influence. The remaining variables are not significant but are consistent with the expected direction.

1. Comparative apple income has the most significant positive impact on the agglomeration of apple production layout, which is consistent with the expected direction of H4, indicating that farmers will compare the economic benefits between different local crops when investing in different agricultural production projects and determine the optimal production plan according to the principle of maximizing income [30]. When the apple income in the region is higher than that of other crops, rational farmers will choose to increase the apple area, which is conducive to increasing the agglomeration degree of regional apple production.
2. Infrastructure, such as water conservancy systems and transportation, has a significant positive impact on the agglomeration of apple production layout, which is consistent with the expected direction of H5 and H6, indicating that irrigation facilities and other facilities are conducive to improving apple production conditions and ensuring the stability of apple production [29, 32]. The accessibility degree of transportation is conducive to increasing the efficiency of apple circulation, facilitating the docking of apple production and sales, and contributing to the realization and enhancement of the market value of apples [30].
3. The policy environment has a significant impact on the agglomeration of apple production layout. Among them, the PGRS has a significant negative impact on the agglomeration of apple production layout, which is consistent with the expected direction of H9, indicating that the PGRS and other food security policies have promoted the continuous expansion of grain area and produced an extrusion effect on apple production [38, 39]. Apple advantageous regional planning policy has had a significant positive impact on the agglomeration of apple production layout, which is consistent with the expected direction of H10, indicating that apple advantageous regional planning guided apple production to the Loess Plateau and Bohai Bay dominant areas and was conducive to the agglomeration of apple production layout [18].
4. The areas of production affected by disasters have a significant positive impact on the agglomeration of apple production layout, which is inconsistent with the expected direction of H1. On the one hand, as a result of upgrading and promotion of orchard equipment and infrastructure, the prevention and mitigation ability of apple disaster has increased, and the negative impact of natural disasters on apple production has been minimized [18, 50], which is not a key factor affecting farmers’ apple cultivation [30]. On the other hand, although the disaster probability of apple production in non-appropriate and sub-suitable areas is high, the spill-over effect of the higher relative price of apples in the region, the policy support of industrial poverty alleviation, and the heterogeneity of farmers’ individual rationality induced an increase in the proportions of apple area in the unsuitable and sub-suitable areas, which in turn caused the apple disaster area to have a positive impact on the agglomeration of apple production layout.

## Conclusions and policy implications

This study used descriptive statistical analysis and spatial econometric analysis to quantitatively reveal the evolution trend, agglomeration characteristics, and influencing factors of China’s apple production spatial layout from 1978 to 2016. The results showed the following. (1) Since 1978, China’s apple production has shown a volatile growth trend, which has experienced five stages: stable period, rapid increase period I, rapid increase period II, rapid decline period, and slow increase period. (2) The centre of apple production in China has moved to the southwest part of the country (the advantage area of the Loess Plateau surpassed the advantage area of the Bohai Bay to become the largest apple production region in China). The concentrations of apple production have increased in 13 provinces and decreased in 9 provinces. (3) Apple production showed significant spatial autocorrelation, but its spatial agglomeration decreased. The hotspot regions of the apple area evolved from Hebei and Henan in 1978 to Shanxi and Henan in 2016, and the main apple-producing areas have gradually shifted from the eastern region to the central region. (4) Comparative apple income, apple effective irrigation area, transportation density, “provincial governors assuming responsibility for the rice bag”, apple advantage regional planning, and apple disaster area are the main factors affecting the agglomeration of apple production layout. Apple industry agglomeration is the key to stabilizing the supply in apple products and ensuring the safety of the apple industry. It is also an inevitable trend in the development of the apple industry. Timely grasping the evolution trend of the centralization production level and agglomeration characteristics of the apple industry is an important premise to scientifically optimize the production layout of the apple industry and is also an important content to steadily advance the regional division of labour and specialization and to stabilize and ensure the adequate supply of apple products.

Based on the above conclusions, the following policy implications were drawn. First, based on the supply-side structural reform fundamentals, it is necessary to improve the quality and profit of apples. The comparative income of apples is the basis for decision-making by farmers to grow apples and is also the scientific principle for promoting the concentration of apple production in the region. Under the realistic background of the transformation of the apple industry from quantitative to qualitative and upgrading the apple’s consumer market demand, the government should improve the quality and market value of apples based on the regional comparative advantages, strengthen spatial agglomeration in advantageous regions (such as the Loess Plateau production area), and build some modern apple production bases to increase economic benefits. Second, as infrastructure development is the basis for ensuring the production and value of apples, it is necessary to pay more attention to the construction of farmland water conservancy facilities, agricultural product transportation, and supporting facilities to improve the production efficiency and product circulation. Third, under the policy background of supply-side structural reform and rural revitalization strategy, it is imperative to accurately grasp the evolution patterns of apple production layout and agglomeration characteristics and adhere to the principle of adaptability of resource endowment and production reality. Furthermore, combined with the development need of the apple industry in the new era, it is necessary to formulate a reasonable layout plan for the apple-producing area, regulate the implementation of regional apple industry planning, enhance the apple production in advantageous production regions and strengthen the technological advancement of apple industry agglomeration. Fourth, under the climate change background of frequent natural disasters in apple, construction of new apple orchards must be according to scientific evaluation of the suitability for apple cultivation. At the same time, disaster prevention and mitigation strategies (such as anti-hail net, antifreeze, artificial pollination, and artificial grass) should be implemented to reduce disaster losses and stabilize the apple supply.

Compared with the existing literature, this study more clearly revealed the spatial-temporal dynamics of apple production. However, there are some shortcomings. First, the growth of apple trees is significantly affected by geographical microclimate. In the future, research needs to be conducted on smaller spatial scales, such as county-level and grid units. Resource endowments and polices, especially low-grade units, may affect the apple production of small-scale spatial units. Second, due to data limitations of national statistics, this study did not address the monopoly in the submarkets, which could be defined according to geometric boundaries or type of apple, and failed to use spatial panel data for a more comprehensive analysis. These issues need to be further explored in future research.
